# Identification of Gray Leaf Spot Disease Candidate Gene in Narrow-Leafed Lupin (*Lupinus angustifolius* L.)

**DOI:** 10.3389/fgene.2021.695791

**Published:** 2021-08-05

**Authors:** Gaofeng Zhou, Huaan Yang, Daniel Renshaw, Meilin Zou, Geoff Thomas, Chengdao Li

**Affiliations:** ^1^Department of Primary Industries and Regional Development Western Australia, South Perth, WA, Australia; ^2^Western Crop Genetics Alliance, Murdoch University, Murdoch, WA, Australia

**Keywords:** SNP, lupin, grey leaf spot, QTL, fine mapping, sequencing

## Abstract

Selection for resistance against gray leaf spot (GLS) is a major objective in the lupin breeding programs. A segregation ratio of 1:1 (resistant:susceptible) in F_8_ recombinant inbred lines (RIL_8_) derived from a cross between a breeding line 83A:476 (resistant to GLS) and a wild accession P27255 (susceptible to GLS) indicated that GLS was controlled by a single major gene. To develop molecular markers linked to GLS, in the beginning, only 11 resistant lines and six susceptible lines from the 83A:476 and P27255 population were genotyped with MFLP markers, and three MFLP markers were identified to be co-segregated with GLS. This method was very efficient, but the markers were located outside of the gene, and could not be used in other germplasms. Then QTL analysis and fine mapping were conducted to identify the gene. Finally, the gene was narrowed down to a 241-kb region containing two disease resistance genes. To further identify the candidate gene, DNA variants between accessions Tanjil (resistant to GLS) and Unicrop (susceptible to GLS) were analyzed. The results indicated that only one SNP was detected in the 241 kb region. This SNP was located in the TMV resistance protein N-like gene region and also identified between 83A:476 and P27255. Genotyping the Tanjil/Unicrop RIL_8_ population showed that this SNP co-segregated with GLS resistance. The phylogenetic tree analysis of this gene among 18 lupin accessions indicates that Australian resistant breeding line/varieties were clustered into one group and carry two resistant alleles, while susceptible accessions were clustered into different groups.

## Introduction

Molecular markers linked to genes of agronomic traits can be applied in plant breeding to hasten genetic improvement. Marker-assisted selection (MAS) requires that the markers should be cost effective, reliable, and be applicable in high-throughput systems for many samples. In addition, the marker genotypes must be consistent with the plant phenotypes across a diverse range of germplasm to enable wide application in breeding programs. Unfortunately, most molecular markers are developed from the outside of the gene region; in these cases, new recombination can happen between the gene and the marker in plant breeding programs. As a result, cultivars exhibiting the ideal marker genotypes may not necessarily carry the targeted genes, which produce “false positive” (Sharp et al., 2001; Boersma et al., 2009). Due to the occurrence of “false positives” or “false negatives” in MAS practices, breeders must validate markers to determine which markers suit which crosses in breeding populations (Eagles et al., 2001; Sharp et al., 2001). This marker validation step can slow down the pace of MAS implementation and increase the cost in breeding programs. A key limiting factor of MAS in plant molecular breeding is to develop diagnostic markers that are applicable for a wide range of germplasm (Brown-Guedira, 2005). The best solution to the plight of a false result in MAS is to develop “diagnostic markers,” which have their genotypes consistent with their phenotypes. Diagnostic markers can be applied for MAS in breeding programs without the need of a marker validation step (Ogbonnaya et al., 2001). For functional genes where gene sequences are available, diagnostic PCR markers can be designed based on polymorphic sites within gene sequences (Zhou et al., 2013). For agronomic traits without the knowledge of gene sequences, one of the methodologies for developing widely applicable molecular markers is to design several markers linked to the target genes, followed by a validation step to test the candidate markers and select the best one for MAS (Yang et al., 2008). Using this concept, diagnostic markers have been developed and applied for anthracnose disease resistance (You et al., 2005) and phomopsis stem blight disease resistance (Yang et al., 2013a) selection in Australian national lupin breeding programs. However, the development of several markers associated with the genes of interest by gel-based DNA fingerprinting methods is very tedious and time consuming. Recently, the rapid advancement and the reduction in the cost of the next-generation sequencing technology (NGS) have made it feasible for many molecular laboratories to embrace the genome sequencing wave. Whole-genome sequencing is the ultimate approach to generate genome-wide, maximum number of molecular markers in plant species. For instance, over 55 million SNPs (single-nucleotide polymorphisms) were detected by genome sequencing and re-sequencing in maize (Chia et al., 2012; Jiao et al., 2014). Therefore, the whole-genome sequencing approach offers tremendous potential for developing diagnostic markers and/or identifying genes in plant breeding.

**FIGURE 1:**
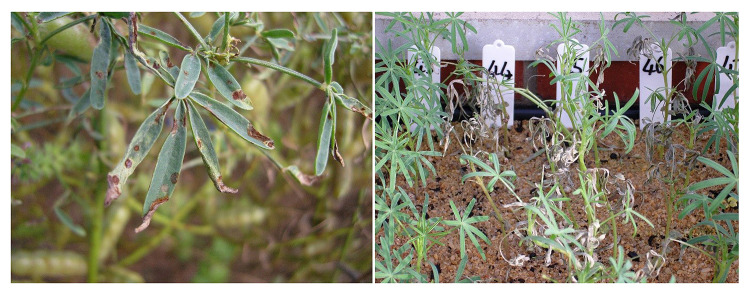
Gray spot leaf disease and phenotyping in glasshouse. Left: disease in adult plant; Right: disease assessment in glasshouse at the seeding stage.

Narrow-leafed lupin (*Lupinus angustifolius* L.) was fully domesticated as a grain legume crop during the 1960s in Australia. Pedigree records showed that 31 varieties were released from 1967 to 2016. Lupin is a grain legume crop and provides a source of protein for animals and humans. Gray leaf spot (GLS) is a serious disease in narrow-leafed lupin (*Lupinus angustifolius* L.), caused by the fungal pathogens *Stemphylium botryosum* spp. (Vaghefi et al., 2020). It causes leaf lesions and defoliation (Figure 1) as well as infecting stems and pods. Dark-brown circular lesions can often appear on leaves and then progress to ash-brown necrosis, and in severe cases, plants lose all their leaves, which can lead to significant yield loss (Thomas et al., 2011). Genetic studies on three separate breeding populations in North America revealed that the resistance to GLS was regulated by a single gene (Forbes et al., 1961). The first three commercial cultivars (Uniwhite, Uniharvest, and Unicrop) released in Australia before 1974 were susceptible to GLS. A collaborative breeding effort between USDA and the Department of Agriculture in Western Australia resulted in the introduction of the gray leaf spot-resistant gene into the Australian germplasm and the release of the first resistant variety in 1974. This resistant (R) gene had been integrated into all the subsequent commercial cultivars over the following 30 years (Thomas et al., 2011). The R gene *gl* is so effective against the disease that it has been effectively absent from Australian lupin crops for over 30 years; during that time, resistance screening for GLS was not carried out by the lupin breeding program. However, GLS re-emerged in Western Australia in 2006. Subsequent disease screening tests in 2008 and 2009 found that approximately 40% of the breeding lines in the Australian lupin breeding program were susceptible to GLS (Thomas et al., 2011). Since then, screening and selection for GLS has become an essential component of the program. The gel-based DNA fingerprinting method MFLP (microsatellite fragment length polymorphism) (Yang et al., 2001, 2002) has been applied in marker development for anthracnose and phomopsis resistance in lupin.

In this study, first, we used an efficient method and identified molecular markers linked to GLS by genotyping only 17 RIL lines. Second, we fine mapped the QTL region and identified the candidate gene for GLS using RIL_8_ populations, and then an SNP diagnostic marker was developed within the gene region, and genotyping RIL population confirmed that 83A:476 and Tanjil shared the same QTL/locus. Finally, GLS alleles were investigated in 18 Australian varieties and European wild ecotypes, and a phylogenetic tree was constructed.

## Materials and Methods

### Plant Materials

Two populations of F_8_ recombinant inbred lines (RIL) were used in this study. They were developed using a single seed descent from their F2 generations. The population (109 RILs) derived from a cross between the Australian domesticated breeding line 83A:476 (resistant to GLS) and Morocco wild-type P27255 (susceptible to GLS) was used for genetic map construction and QTL mapping for GLS confirmation. Another 93 RIL_8_ population derived from the cross between two Australian cultivars Tanjil (resistant to GLS) and Unicrop (susceptible to GLS) was used to validate the QTL and the molecular markers in this study. Unicrop was one of the three susceptible varieties released prior to the introduction of GLS resistance, while Tanjil was released in 1998 as part of the lineage with the GLS resistance. A total of 18 historical and current commercial cultivars and breeding or wild ecotypes (Tanjil, Yorrel, Coromup, Mandelup, Merrit, 83A:476, Moonah, Unicrop, Kalya, Tallerack, P26167, P27255, Quillinock, 75A:258, P26603, Bo7212, P26668, and P27221) were used to analyze GLS alleles and the gene evolution. These germplasms are from Australian varieties and European wild ecotypes.

### Phenotyping Gray Leaf Spot Resistance

Two isolates (*S. beticola*: WAC12986 and *S. vesicarium*: WAC13136) from the Western Australian Culture Collection (Thomas et al., 2011; Vaghefi et al., 2020) were used for phenotyping in this study. Isolates were grown on 20% V8 agar medium at 22°C with a 12:12 h light/dark cycle for 14 days (Everts and Armentrout, 2001). Then spores were harvested and suspended in sterile distilled water with 0.1% Tween-20. The final concentration of spores was adjusted to 1.5 × 10^5^ ml^–1^. Three replicate pots, each containing seven 14-day-old seedlings of each line were spray inoculated with the spore suspension. The pots were placed randomly. Inoculated plants were placed in an intermittent misting growth chamber with 90% of shade of natural daylight. Two days later, the plants were transferred to the greenhouse at 20°C. Disease scoring was conducted 14 days after inoculation. Disease resistance was scored on the first two fully expanded leaves on a 0–5 scale (0: no symptoms; 1: one to two lesions < 1 mm on each leaflet; 2: less than five small lesions on each leaflet; 3: 1- to 5-mm lesions, often coalescing with associated chlorosis; 4: some leaflets completely necrotic or fallen; 5: all leaflets completely necrotic or complete defoliation). Plants scoring 0 and 1 were considered resistant, while those scoring greater than 3 were considered susceptible (Thomas et al., 2011; Ahmad et al., 2016). Genotype effects on disease severity were analyzed by ANOVA with differences compared by LSD. The disease assessments of the two RIL populations were carried out in 2008, 2009, and 2013.

### Identification of Markers Linked to Gray Leaf Spot Resistance by Microsatellite Fragment Length Polymorphism

Identification of MFLP markers linked to GLS resistance followed the same method as described by previous studies (Yang et al., 2002; Lin et al., 2009). MFLP method combines the concept of microsatellite-anchor primer and AFLP technique. Genomic DNA was digested by restriction enzyme *Mse*I. One AFLP *Mse*I adaptor was ligated onto the restriction fragments. PCR was performed using one *Mse*I primer in combination with one microsatellite-anchor primer (Yang et al., 2001). Seventeen plants were used in the MFLP analysis. Eleven of these plants, including parental line 83A:476 and 10 RIL_8_, were resistant to GLS. The other six plants, including parental line P27255 and five RIL_8_, were susceptible to GLS. These plants were genotyped with MFLP markers. The genotypes of the markers close to GLS should be associated with the disease resistance.

### Sequencing Microsatellite Fragment Length Polymorphism Amplicons

PCR bands from the MFLP markers linked to GLS were excised from MFLP gels and boiled for 1 min in TE buffer. The products were re-amplified by PCR, and electrophoresed in 1% agarose gel. DNA fragments were isolated with agarose gel and purified with a gel extraction kit (Qiagen), and then sequenced using the BigDye Terminator system (Applied Biosystems). Blastn searches were performed on the sequences in the lupin genome database (Wang et al., 2020). The chromosome and physical positions of MFLP markers were anchored.

### Sequencing

The lupin parental lines (Unicrop, 83A:476, and P27255) were shotgun sequenced with 10 times coverage using the second-generation sequencing method, and the sequences were obtained from the China National GeneBank Database^1^ and were available under the project accession CNP0001034. The RIL_8_ population from the cross of 83A:476 and P27255 were genotyped using the RAD method (Yang et al., 2013b; Zhou et al., 2018). The protocol was described by Chutimanitsakun et al. (2011) with minor modifications that *Eco*RI was used. Sequencing libraries (100 bp) were constructed by using a unique multiplex identifier (MID) barcode (Baird et al., 2008). The RAD product sequencing was carried out on Solexa HiSeq2000. Sequencing raw data were divided into different lupin lines according to the MID barcodes (Baird et al., 2008), and then the MID barcodes were removed from the raw sequencing data. The 92-bp RAD reads with the same DNA sequences were clustered into one read tag. Tags containing over 100 reads were considered to be from repetitive regions, so they were removed to avoid the detection of SNP/indel variants (Catchen et al., 2011). Genome assembly of the two parental lines was conducted before alignment.

### QTL Mapping and Fine Mapping

MapQTL5.0 was used to detect QTL for gray leaf spot disease. The genetic map of the population between 83A:476 and P27255 was obtained from our previous studies (Zhou et al., 2018). Formatted files including the genetic map, genotypic data, and phenotypes were imported into MapQTL5.0. The threshold of LOD value for QTL mapping was 3.0. First, interval mapping was performed, and then the marker with the highest LOD value was selected for MQM mapping analysis.

High-density markers across the QTL region were genotyped in the RIL population. The recombinant lines were selected to narrow down the QTL for gray leaf spot resistance. The left border and right border of the gene were determined by comparing the genotypes and phenotypes.

### Variant Detection

The software CLC Genomic Workbench 21.0.3 was used to map the sequence data to the reference narrow-leafed lupin “Tanjil” genome. The 17 accessions excluding the reference cv. Tanjil were shotgun sequenced with 10 times coverage. All the sequencing read data were obtained from the China National GeneBank Database (see text footnote 1) and were available under the project accession CNP0001034. The parameters were as follows: match score 1, mismatch cost 2, linear gap cost, length fraction 0.5, similarity fraction 0.8, and non-specific match handling with map randomly. Tracks were created, and all the genome sequences used in this study were combined. The SNP and indel variants were detected and generated. For variant detection, fixed ploidy variant parameters were as follows: ploidy 2 and required variant probability 90%, ignore broken pairs, minimum coverage 10, minimum count 2, and minimum frequency 20%.

### Phylogenetic Tree Construction

Gray leaf spot (GLS) alleles and gene evolution were investigated among 18 narrow-leafed lupin accessions. SNPs were identified by mapping their reads to the Tanjil reference genome. SNP variants were obtained from the variants detection. The SNP genotype data were imported into Geneious 8.0 for phylogenetic tree construction. Geneious Tree Builder function was used, and the parameters were as follows: genetic distance model of Jukes–Cantor with the build method of UPGMA.

## Results

### Single Gene Controls Gray Leaf Spot Resistance

**FIGURE 2:**
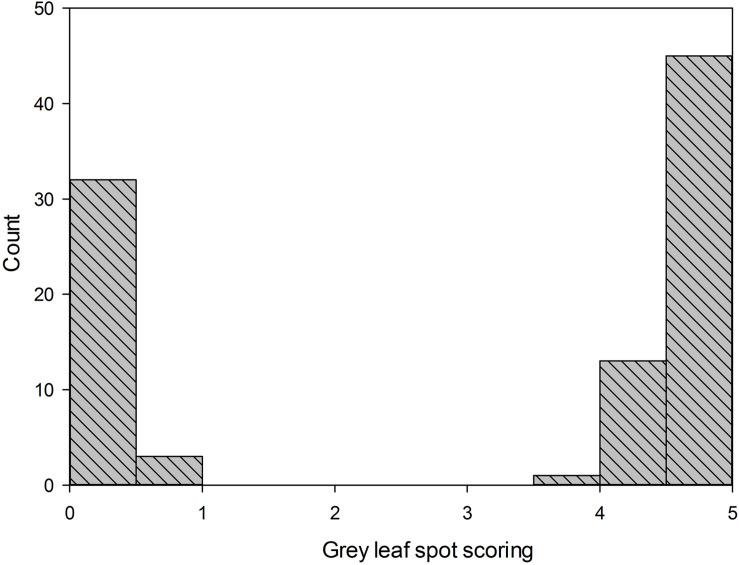
Distribution of gray leaf spot scoring in F8 recombinant inbred lines (RIL_8_) population. Line 83A:476 score was 0 (resistant), and wild-type P27255 was 4.65 (susceptible); *n* = 111.

For the gray leaf spot disease phenotypes, all the plants of parental line 83A:476 were resistant to GLS (scoring 0), while all the plants of P27255 were susceptible (scoring 4 or 5, mean 4.6). The parental phenotypes were significantly different (*p* < 0.05). Among the 109 tested F_8_ RILs, 51 RILs were susceptible to GLS, and the other 58 RILs were resistant (Figure 2). The segregation of susceptible:resistant in the F_8_ population fit the expected 1:1 ratio (*χ*^2^ = 0.33, *χ*^2^_0_._05_,_1_ = 3.84, (χ^2^ < χ^2^_0_._05_,_1_), indicating that a single gene controls GLS resistance in 83A:476.

### Identification of Microsatellite Fragment Length Polymorphism Markers Linked to Gray Leaf Spot Resistance

In the beginning, we wanted to develop molecular markers linked to GLS and use immediately in breeding programs, so an efficient method was adopted to identify the locus. A total of 1,657 polymorphic MFLP markers were identified between parental lines 83A:476 and P27255. These markers were genotyped in 10 resistant RILs and 5 susceptible RILs as well. Among these markers, three markers (D145, D280, and D300) co-segregated with GLS resistance in these RILs (Table 1). All the resistant RIL_8_ exhibited the same genotype to 83A:476, and the susceptible RILS showed the same genotypes to P27255.

**TABLE 1:** Microsatellite fragment length polymorphism (MFLP) markers linked to gray leaf spot (GLS) resistance.

Lines	Source	Marker-D145 (LG-19 3.097 Mb)	Marker-D280 (LG-19 3.033 Mb)	Marker-D300 (LG-19 3.033 Mb)	GLS score
83A:476	Parent 1	+	–	–	0.0
Line13	RILs	+	–	–	0.0
Line14	RILs	+	–	–	0.36
Line19	RILs	+	–	–	0.0
Line20	RILs	+	–	–	0.0
Line35	RILs	+	–	–	0.0
Line49	RILs	+	–	–	0.0
Line74	RILs	+	–	–	0.0
Line81	RILs	+	–	–	0.0
Line95	RILs	+	–	–	0.0
Line105	RILs	+	–	–	0.0
P27255	Parent 2	–	+	+	4.6
Line28	RILs	–	+	+	4.9
Line29	RILs	–	+	+	4.9
Line70	RILs	–	+	+	4.6
Line87	RILs	–	+	+	4.8
Line88	RILs	–	+	+	4.7

### Sequencing of Linked Microsatellite Fragment Length Polymorphism Markers and Anchor to Physical Map

The PCR products from PAGE gel were amplified and sequenced. The amplicon sequences of the three markers are listed in Supplementary Table 1. The markers did not perform very well (data not shown) among the lupin germplasms maybe because these markers were located outside of the GLS gene, so we continued to identify the gene and develop diagnostic markers. Recently, the release of the lupin genome sequence enables us to anchor the MFLP markers to their physical positions and to mine the genes based on the lupin genome (Wang et al., 2020). The sequences excluding forward and reverse primers were used to conduct a blast in the lupin cv. Tanjil genome. All the three markers (D145, D280, and D300) were anchored to the lupin linkage group LG-19 with a physical position of 2.156–2.218 Mb region. D280 and D300 were anchored to the 2.156 Mb region, while D145 was mapped to the 2.218 Mb region.

### QTL Mapping and Fine Mapping

**FIGURE 3:**
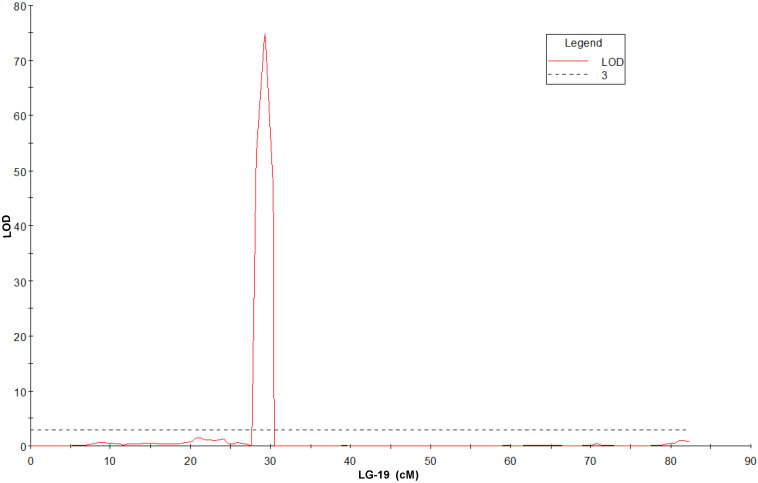
QTL for gray leaf spot disease resistance on LG-19 in narrow-leafed lupin (LOD = 3).

QTL mapping and fine mapping were used to validate the candidate gene. The genetic map from 83A:476 and P27255 population was constructed in our previous studies (Zhou et al., 2018). The genetic map contains 27,892 markers (Supplementary Data Sheet 1). A simplified map containing one marker for each locus was used for QTL mapping. QTL analysis was conducted in the RIL population. A single major QTL for gray leaf spot resistance was identified on chromosome 19. The closest marker DAFWAsnp_16977 (29.33 cM) can explain 97.9% phenotypic variations and with the LOD value of 74.83 (Figure 3 and Supplementary Figure 1).

**FIGURE 4:**
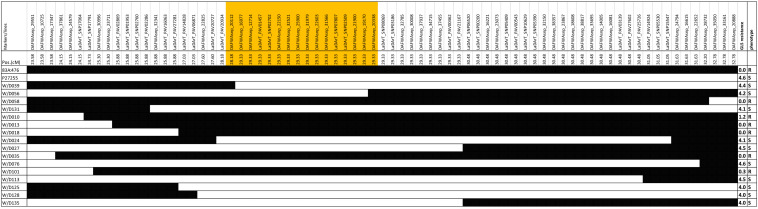
Recombinant lines for fine-mapping of gray leaf spot resistance.

The recombinant lines around the QTL region (23.58 to 32.78 cM) were investigated in the RIL_8_ population. A total of 16 recombinant lines were obtained based on the genotypes (Figure 4). Figure 4 showed that the genotypes of QTL marker DAFWAsnp_16977 were consistent with their gray leaf spot resistance in this population. From these recombinant lines, the gene was narrowed down by the lines W/D039 and W/D056. The recombination point of W/D039 was between the markers DAFWAsnp_20112 (R genotype) and DAFWAsnp_16977 (S genotype), while the GLS resistance of this line was susceptible, so the gene must be on the right side of the marker DAFWAsnp_20112. For the line W/D056, the recombination point was between the markers DAFWAsnp_19170 (S genotype) and DAFWAsnp_20338 (R genotype), and the GLS resistance was susceptible, so the gene should be on the left side of the marker DAFWAsnp_20338. In a word, the gene was narrowed down between the two markers DAFWAsnp_20112 and DAFWAsnp_20338. These markers were anchored to narrow-leafed lupin cv. Tanjil genome, and the physical positions of the markers were 2,157,779 and 2,399,114 bp, respectively. The QTL was finally fine-mapped to a 241-kb region. The three MFLP markers (D145, D280, and D300) overlapped with this fine mapping region.

### Genes in the Region

A total of 21 genes were annotated in the QTL mapping region (Table 2). Two genes, LOC109334326 and LOC109334327, were associated with disease resistance. LOC109334326 is a TMV resistance protein N-like, and LOC109334327 is a putative disease resistance protein RGA3 (Wang et al., 2020).

**TABLE 2:** List of annotated genes in the fine-mapping region.

Lupin gene ID	Annotated ID	Description
XM_019569076.1	LOC109333574	Probable sugar phosphate/phosphate translocator At5g04160
xm_019569260.1	LOC109333709	Uncharacterized
XM_019569303.1	LOC109333739	Uncharacterized
XM_019569319.1	LOC109333752	Zinc finger protein WIP2-like
XM_019569320.1	LOC109333754	Syntaxin-121-like
xm_019569325.1	LOC109333759	Zeatin O-glucosyltransferase-like
XM_019569329.1	LOC109333763	Remorin
XM_019569426.1	LOC109333847	Mulatexin
xm_019569492.1	LOC109333910	Uncharacterized mitochondrial protein AtMg00810-like
XM_019569493.1	LOC109333911	F-box/kelch-repeat protein At3g23880-like
XM_019570034.1	LOC109334326	TMV resistance protein N-like
XM_019570035.1	LOC109334327	Putative disease resistance protein RGA3
xm_019570323.1	LOC109334506	Uncharacterized
XM_019570326.1	LOC109334508	Uncharacterized
XM_019570390.1	LOC109334557	Adenylosuccinate synthetase 2, chloroplastic
xm_019570483.1	LOC109334615	1-acyl-sn-glycerol-3-phosphate acyltransferase 2
XM_019570535.1	LOC109334656	DNA-binding protein SMUBP-2
XM_019570536.1	LOC109334658	Dehydration-responsive element-binding protein 2F
XM_019570540.1	LOC109334660	Uncharacterized
xm-019570782.1	LOC109334820	Guanylate kinase 2
XM_019570938.1	LOC109334924	Calcium-dependent protein kinase 20

### Candidate Gene Identification

**FIGURE 5:**
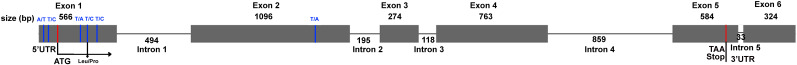
Candidate gene LOC109334326 DNA structure. Introns and exons are represented with continuous lines and gray rectangular boxes. Start codon and stop codon are marked with red lines. 5’-UTR and 3’-UTR are shown in the figure. SNP variations are marked with blue lines and letters (83A:476 on the left, and P27255 on the right). One amino acid substitution is indicated in the figure.

Genomic sequence analysis revealed that there were six SNPs in the LOC109334326 region between 83A:476 and P27255 (Figure 5). Two SNPs were in the 5′-UTR regions, while the other four SNPs were in the coding region. Only one SNP (T/C) at 2,190,169 in the coding region induces amino acid substitution from leucine in 83A:476 (R) to proline in P27255 (S) (Figure 5).

To further investigate these two genes, we used two additional Australian cultivars Tanjil and Unicrop. Tanjil is resistant to gray leaf spot disease, while Unicrop is susceptible. Unicrop was re-sequenced and mapped to the Tanjil reference genome. The segregation of susceptible:resistant in the F_8_ population fit the expected 1:1 ratio (*χ*^2^ = 3.76, χ^2^_0_._05_,_1_ = 3.84), (χ^2^ < χ^2^_0_._05_,_1_). It indicated that a single gene controlled GLS resistance in the Tanjil and Unicrop population. Interestingly, the result indicated that there was only one SNP variation from T (Tanjil) to C (Unicrop) at 2,190,196 bp in the 241-kb fine-mapping region on chromosome 19, and this SNP variation from the gene LOC109334326 results in the same amino acid substitution from leucine in Tanjil (R) to proline in Unicrop (S). It indicates that this is the candidate gene for gray leaf spot resistance.

For another disease resistance RGA3 gene LOC109334327, 17 SNPs were detected between the parental lines 83A:476 and P27255 (Table 3), but no variations exist between Tanjil and Unicrop. Furthermore, no SNPs were identified between the tolerant line 83A:476 and the susceptible cultivar Unicrop. It indicates that this gene is not the candidate gene.

**TABLE 3:** Variations in the gene LOC109334327 region.

Mapping	Position (bp)	Length (bp)	83A:476 (R)	P27255 (S)	Unicrop (S)
LG19	2178851	1	C	T	C
LG19	2179252	2	CT	GG	CT
LG19	2179279	1	A	G	A
LG19	2179290	1	G	C	G
LG19	2179876	1	A	G	A
LG19	2179880	1	G	C	G
LG19	2180038	1	T	G	T
LG19	2180046	1	T	G	T
LG19	2180445	1	T	C	T
LG19	2180462	1	G	A	T
LG19	2180774	1	G	A	G
LG19	2180780	1	T	C	T
LG19	2181125	1	T	C	T
LG19	2181150	1	G	T	G
LG19	2181221	1	T	C	T
LG19	2181386	1	A	G	A
LG19	2181491	1	T	G	A

### Single-Nucleotide Polymorphism Marker Development for Gray Leaf Spot Resistance

To confirm the Tanjil carrying the same GLS QTL/locus, which was mapped from 83A:476 and P27255 population, a dominant SNP resistant marker was developed for the GLS candidate gene (forward: TCTGATGGCGTACATGTGTAA, reverse: TACTCTGCCCTCACTGACCT). This marker was tested in 93 RIL_8_ derived from a cross between Tanjil and Unicrop. The genotypes co-segregated with their GLS resistance (Supplementary Table 2).

### Phylogenetic Tree

**FIGURE 6:**
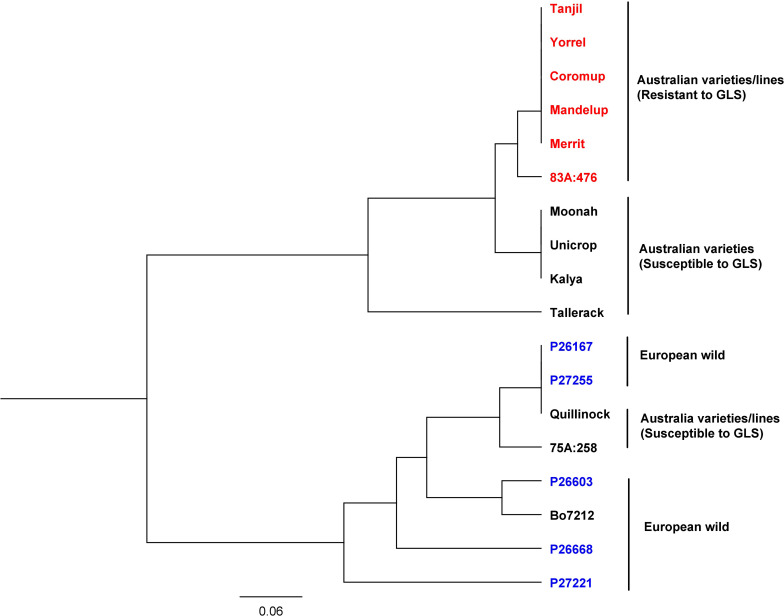
Phylogenetic tree of gray leaf spot candidate gene from 18 narrow-leafed lupin accessions.

The gene alleles were investigated among Australian and European lines. SNP variants of the gene (LOC109334326) were analyzed among 18 narrow-leafed lupin accessions consisting of Australian varieties, Australian breeding lines, and European wild lupin (Tanjil, Yorrel, Coromup, Mandelup, Merrit, 83A:476, Moonah, Unicrop, Kalya, Tallerack, P26167, P27255, Quillinock, 75A:258, P26603, Bo7212, P26668, and P27221). The genomic sequences of these accessions were obtained from the China National GeneBank Database (see text footnote 1) and were available under the project accession CNP0001034. The sequence reads were mapped to the reference genome Tanjil, and SNP variants were obtained (see Supplementary Data). The phylogenetic tree (Figure 6) showed that they were classified into two major groups. All the European wild lupin accessions, together with the Australian variety/breeding lines Quillinock and 75A:258, were in one group, while all the other Australian varieties/breeding lines were clustered into another group. Furthermore, all the resistant (Tanjil, Yorrel, Coromup, Mandelup, Merrit, and 83A:476) and susceptible varieties (Moonah, Unicrop, Kalya, and Tallerack) from the major group were divided into different sub-groups. For the resistant gene, two alleles were identified. Australian resistant cultivars Tanjil, Yorrel, Coromup, Mandelup, and Merrit carried the same allele, while the breeding line 83A:476 carried a different GLS allele. It indicated that new GLS-resistant allele from lupin germplasm was incorporated into the breeding line 83A:476 during the breeding program.

## Discussion

In the present study, the rapid mapping method was used to develop markers for the GLS resistance gene in lupin. Based on the GLS segregation ratio of 1:1 in 109 RILs, it was confirmed that only one major resistance locus existed in this population. At the first step, only 15 RILs from resistance and susceptible groups were selected for gene mapping. This method significantly reduced cost and saved time. Eleven resistant lines and six susceptible lines were genotyped with MFLP markers. A total of 1,657 polymorphic markers were identified in the RILs. Finally, three co-segregated MFLP markers (D145, D280, and D300) were identified.

The sequences of the three MFLP amplicons were obtained and anchored to the lupin linkage group LG19 with the physical position of 2.156 and 2.218 Mb by running blast against the lupin cv. Tanjil reference genome (Wang et al., 2020). It indicated that the three MFLP were in the same chromosome and the same region in lupin.

To compare the result with the QTL mapping result. The bio-parental lines were re-sequenced using a RAD method. A major advantage of RAD sequencing is that it can generate tens of thousands of SNPs and InDels (insertions and deletions), which can be used for MAS in plant-breeding programs. Furthermore, all the variants detected from RAD sequencing have had the DNA sequenced already. Therefore, conversion of variants into PCR-based markers does not need PCR amplification, ligation, transformation, and sequencing as required from traditional markers such as AFLP (Shan et al., 1999), RAPD (Paran and Michelmore, 1993), and MFLP (Yang et al., 2002). One single QTL was detected, and the closest marker DAFWAsnp_16977 was mapped to 2.167 Mb on chromosome LG19. This result is consistent with the positions of the MFLP markers (2.156–2.218 Mb). It indicated that rapid genotyping of a few lines can be an effective and efficient way to develop disease-linked markers if the trait is controlled by a single major gene, and phenotypes are very stable for quantitative traits.

A high-density genetic map and many population lines will facilitate us to narrow down the QTL region. In this study, the QTL was mapped to a 241-kb region. There were two genes (LOC109334326 and LOC109334327) associated with disease resistance. Even though there were 17 variations in LOC109334327 between 83A:476 and P27255 (Table 3), no variations were detected between Tanjil (R) and Unicrop (S) in the gene region including 5′-UTR and 3′-UTR. Furthermore, no variations were identified between the tolerant breeding line 83A:476 and the susceptible variety Unicrop. It indicated that this gene was not associated with gray leaf spot resistance. For the other gene LOC109334326, one SNP variation resulted in one amino acid substitution between 83A:476 (R) and P27255 (S), and this SNP was also the only variation between Tanjil (R) and Unicrop (S) in the 241-kb QTL region based on re-sequence mapping result. We assumed that Tanjil and 83A:476 shared the same gene or locus. After the candidate gene was identified, molecular markers should be used to validate that the gene marker co-segregated with the resistance in the Tanjil and Unicrop population.

A dominant SNP marker for GLS was developed based on the SNP variation in the candidate gene LOC109334326 and validated in the RIL population derived from the cross between Tanjil and Unicrop. The genotypes co-segregated with their phenotypes in the RIL population. It indicated that Tanjil and 83A:476 could have the same gene or locus. Currently, there are several methods for SNP marker development, such as McSNP (melting curve analysis of SNPs), KASP (kompetitive allele-specific PCR), and CAPS (cleaved amplified polymorphic sequences). This dominant marker can be converted to these kinds of markers depending on the laboratory instruments.

Phylogenetic tree results indicated that the resistance allele of GLS candidate gene was different from the alleles from the European wild ecotypes because the resistant allele was found from a United States wild ecotype of *Lupinus angustifolius*, which was later incorporated into the Australian varieties since the 1970s (Thomas et al., 2011). Two resistant alleles indicated that the breeding line 83A:476 incorporated a new allele from our lupin germplasm.

The results demonstrated that RAD sequencing is a rapid approach to identify molecular markers closely linked to the trait of interest for MAS in breeding programs. Whole-genome re-sequencing can facilitate us to identify the DNA variations and candidate genes. The transgenic complementation of the candidate gene and GLS resistance mechanism will be studied in the future.

## Data Availability Statement

The datasets presented in this study can be found in online repositories. The names of the repository/repositories and accession number(s) can be found in the article/Supplementary Material.

## Author Contributions

HY, DR, and GT performed the genotyping and phenotyping. GZ analyzed the data and wrote the manuscript. MZ analyzed the phylogenetic tree. HY and CL supervised this study. All authors have discussed and revised the manuscript.

## Conflict of Interest

The authors declare that the research was conducted in the absence of any commercial or financial relationships that could be construed as a potential conflict of interest.

## Publisher’s Note

All claims expressed in this article are solely those of the authors and do not necessarily represent those of their affiliated organizations, or those of the publisher, the editors and the reviewers. Any product that may be evaluated in this article, or claim that may be made by its manufacturer, is not guaranteed or endorsed by the publisher.

## Footnotes

1
https://db.cngb.org

## Supplementary Material

Supplementary Figure 1 QTL for gray leaf spot disease resistance in narrow-leafed lupin (Threshold LOD = 3).

Supplementary Table 1 MFLP marker amplicon sequence.

Supplementary Table 2 SNP genotyping and GLS phenotyping in RIL population derived from Tanjil and Unicrop. ? represents unclear phenotype.

Supplementary Table 3 SNPs of LOC109334327 in narrow-leafed lupin germplasm.

Supplementary Data Sheet 1 Genotypes and GLS phenotypes in mapping population.
