# Supplementary material for: Identification of Gray Leaf Spot Disease Candidate Gene in Narrow-Leafed Lupin (Lupinus angustifolius L.)
Source: Front Genet. 2021 Aug 5;12:695791. doi: 10.3389/fgene.2021.695791 (PMC8375407; doi:10.3389/fgene.2021.695791)
Supplement: Supplementary Figure 1 — QTL for gray leaf spot disease resistance in narrow-leafed lupin (Threshold LOD = 3). [file Data_Sheet_2.PDF]

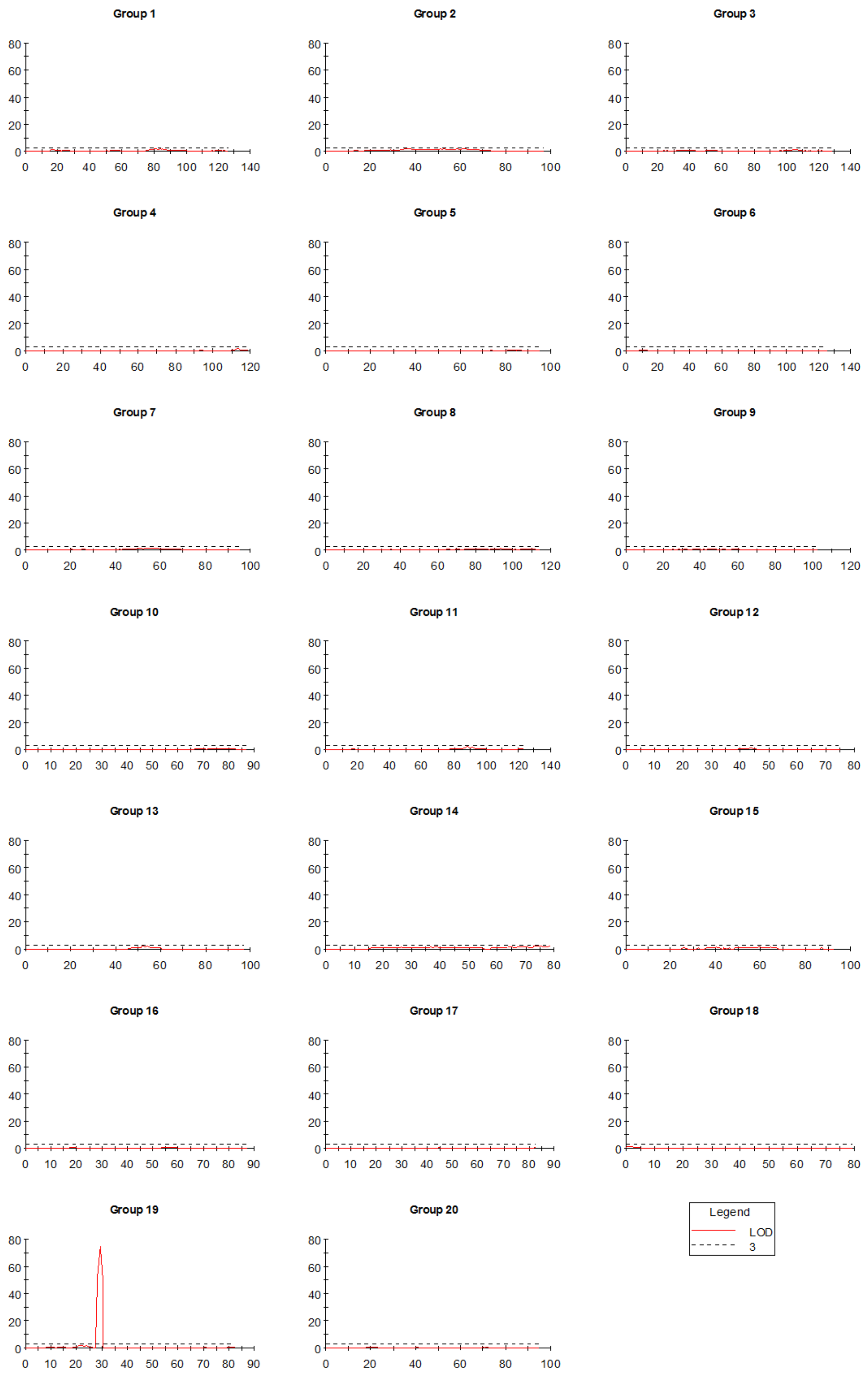

Figure S1 QTL for grey leaf spot disease resistance in narrow-leafed lupin (----: LOD = 3)

Table S1 MFLP marker amplicon sequence

Marker	Sequence
D145	GATGAGTCCTGAGTAAAATAATCAAGATTGTATATATT<br>GAATTGGTCATTTTCTTCCTTCACTTCAGTGCTATTTCT<br>ACTGCATCACTATTTCATGTTTTTTCCTGTTATATTAGT<br>ATGTTTTTCTAAATCCCCTCTCTCTCTCTGCCC
D280	GATGAGTCCTGAGTAAAGTGTAACAATGTTCAAAGAT<br>GTAAATGAAATCCACCTTGATATCCTATTCTATAAATAA<br>CCAACATACCTACCTATTACCACCATCCAAAACACACA<br>TAACCTAAGTCACTTAGTGCTAAATTTCTACAATGCCT<br>TCTAGCTACGAAACTTCACCAATGGCTTCCAATTACAA<br>TAGCCACGAAAAAGTCCATAACAATGGCACCAAAATC<br>TTTCATCAAACCCAAGTAGTTGTTGTTGTTTGGG
D300	GATGAGTCCTGAGTAAAGTGTAACAATGTTCAAAGAT<br>GTAAATGAAATCCACCTTGATATCCTATTCTATAAATAA<br>CCAACATACCTACCTATTACCACCATCCAAAACACACA<br>TAACCTAAGTCACTTAGTGCTAAATTTCTACAATGCCT<br>TCTAGCTACGAAACTTCACCAATGGCTTCCAATTACAA<br>TAGCCACGAAAAAGTCCATAACAATGGCACCAAAATC<br>TTTCATCAAACCCAAGTAGTTGTTGTTGTTGTTAGAC

Table S2 SNP genotyping and GLS phenotyping in RIL population derived from Tanjil and Unicrop. ? represents unclear phenotype.

Lines	GLS rating	Marker genotype
Tanjil	R	R
Unicrop	S	S
2	S	S
7	S	S
8	S	S
16	R	R
19	R	R
20	R	R
22	S	S
23	R	R
24	S	S
26	S	S
27	S	S
28	S	S
29	R	R
32	S	S
33	S	S
37	S	S
38	S	S
39	S	S
40	R	R
41	R	R
42	S	S
46	S	S
47	S	S
51	S	S
53	R	R
54	S	S
55	S	S
57	S	S
59	S	S
61	R	R
63	R	R
65	R	R
66	R	R
68	S	S
70	R	R
71	S	S
76	S	S
79	R	R
80	S	S
82	S	S
86	R	R
89	S	S
91	S	S
96	S	S
98	S	S
100	S	S
102	S	S
103	R	R

113	R	R
122	S	S
126	S	S
128	S	S
135	R	R
158	R	R
160	R	R
162	R	R
164	R	R
166	R	R
167	R	R
168	R	R
173	R	R
174	R	R
204	R	R
208	R	R
211	R	R
225	R	R
137	S	S
146	S	S
149	S	S
155	S	S
157	S	S
170	S	S
176	S	S
178	S	S
184	S	S
189	S	S
196	S	S
200	S	S
212	S	S
213	S	S
218	S	S
221	S	S
226	S	S
201	S	S
216	R	R
222	S	S
131	?	S
163	?	S
177	?	S
209	?	S
233	?	S
248	?	R
249	?	S

Table S3 SNPs of LOC109334327 in narrow-leafed lupin germplasm

Pos.	10	22	33	70	389	410	452	1208	1369	1503	2480	2509	2943	3131	3299	3330
Tanjil	C	T	A	T	T	T	T	T	G	A	T	G	C	G	G	C
Unicrop	C	T	A	T	T	C	T	T	G	A	T	G	C	G	G	C
75A:258	C	T	T	C	A	C	C	T	C	A	A	G	C	G	C	A
P27255	C	T	T	C	A	C	C	T	G	A	A	G	C	G	-	-
83A:476	C	T	A	T	T	T	T	T	G	A	A	G	C	G	-	-
Yorrel	C	T	A	T	T	T	T	T	G	A	-	-	C	G	G	C
Coromup	C	T	A	T	T	T	T	T	G	A	T	G	C	G	-	-
Moonah	C	T	A	T	T	C	T	T	G	A	-	-	C	G	-	-
P26167	C	T	T	C	A	C	C	T	G	A	-	-	C	G	-	-
P26603	C	T	A	C	A	C	C	T	G	A	A	C	C	G	-	-
P26668	C	C	T	C	A	C	C	T	G	A	A	C	A	G	-	-
P27221	T	C	T	C	A	C	C	T	G	G	A	G	C	G	-	-
Tallerack	C	T	A	T	A	C	T	C	G	A	T	G	C	A	G	C
Mandelup	C	T	A	T	T	T	T	T	G	A	-	-	C	G	-	-
Bo7212	C	T	A	C	A	C	T	T	G	A	A	C	C	G	C	C
Quillinock	C	T	T	C	A	C	C	T	G	A	A	G	C	G	-	-
Merrit	C	T	A	T	T	T	T	T	G	A	T	G	C	G	G	C
Kalya	C	T	A	T	T	C	T	T	G	A	T	G	C	G	G	C
